# The effect of intramuscular interferon beta-1a on spinal cord volume in relapsing-remitting multiple sclerosis

**DOI:** 10.1186/s12880-016-0158-4

**Published:** 2016-10-05

**Authors:** Sheena L. Dupuy, Fariha Khalid, Brian C. Healy, Sonya Bakshi, Mohit Neema, Shahamat Tauhid, Rohit Bakshi

**Affiliations:** 1 Department of Neurology, Brigham and Women’s Hospital, Laboratory for Neuroimaging Research, Partners MS Center, Harvard Medical School, Boston, MA USA; 2 Department of Radiology, Brigham and Women’s Hospital, Laboratory for Neuroimaging Research, Partners MS Center, Harvard Medical School, Boston, MA USA; 3 Laboratory for Neuroimaging Research, One Brookline Place, Brookline, MA 02445 USA

**Keywords:** Multiple sclerosis, MRI, Spinal cord atrophy, Interferon beta-1a

## Abstract

**Background:**

Spinal cord atrophy occurs early in multiple sclerosis (MS) and impacts disability. The therapeutic effect of interferon beta-1a (IFNβ-1a) on spinal cord atrophy in patients with relapsing-remitting (RR) MS has not been explored.

**Methods:**

We retrospectively identified 16 consecutive patients receiving weekly intramuscular IFNβ-1a for 2 years [baseline age (mean ± SD) 47.7 ± 7.5 years, Expanded Disability Status Scale score median (range) 1.5 (0–2.5), timed 25-foot walk 4.6 ± 0.7 seconds; time on treatment 68.3 ± 59.9 months] and 11 sex- and age-matched normal controls (NC). The spinal cord was imaged at baseline, 1 and 2 years later with 3T MRI. C1-C5 spinal cord volume was measured by an active surface method, from which normalized spinal cord area (SCA) was calculated.

**Results:**

SCA showed no change in the MS or NC group over 2 years [mean annualized difference (95 % CI) MS: −0.604 mm^2^ (−1.352, 0.144), *p* = 0.106; NC: −0.360 mm^2^ (−1.576, 0.855), *p* = 0.524]. Between group analysis indicated no differences in on-study SCA change [MS vs. NC; year 1 vs. baseline, mean annualized difference (95 % CI) 0.400 mm^2^ (−3.350, 2.549), *p* = 0.780; year 2 vs. year 1: −1.196 mm^2^ (−0.875, 3.266), *p* = 0.245; year 2 vs. baseline −0.243 mm^2^ (−1.120, 1.607), *p* = 0.712].

**Conclusion:**

Established IFNβ-1a therapy was not associated with ongoing spinal cord atrophy or any difference in the rate of spinal cord volume change in RRMS compared to NC over 2 years. These results may reflect a treatment effect. However, due to sample size and study design, these results should be considered preliminary and await confirmation.

## Background

Multiple sclerosis (MS) is a disease of the CNS characterized by lesions and atrophy in both the brain and spinal cord [1]. Measurement of spinal cord atrophy is of growing interest due to improving MRI technology, regarding both scan acquisition and segmentation techniques, facilitating its quantification [2–7]. In addition, a myriad of studies have shown that such atrophy occurs early in the disease course and is a proposed contributor to neurologic disability [1, 5, 8]. Despite the availability of more than 10 disease-modifying immunotherapies for the treatment of MS, few studies have assessed therapeutic effects on spinal cord atrophy [8–14]. Such a pursuit might have relevance in complementing the information on disease severity and treatment effects obtained from brain imaging. In support of this concept, spinal cord metrics provide a unique contribution to brain metrics in modeling the relationship between MRI and clinical status in MS [15]. In addition, a growing body of evidence indicates that spinal cord involvement may occur and progress independently from brain involvement [8, 16–19].

Interferon β-1a (IFNβ-1a), given intramuscularly each week, is an approved MS immunotherapy that has been shown to limit relapse rate, delay the time to a sustained increase in physical disability, and limit cerebral MRI-defined lesion activity and burden of disease in patients with relapsing forms of the disease [20–22]. In addition, studies have indicated the ability of weekly intramuscular IFNβ-1a to limit the rate of brain atrophy [23, 24]. However, no studies to date have examined spinal cord atrophy treatment effects in patients with relapsing forms of MS receiving weekly intramuscular IFNβ-1a. We performed a pilot study to assess the 2 year change in spinal cord volume associated with established IFNβ-1a treatment in comparison to healthy subjects.

## Methods

### Subjects

Baseline demographic and clinical data of the MS and normal control (NC) groups are summarized in Table 1. We retrospectively analyzed 16 consecutive patients with relapsing-remitting MS (RRMS) receiving established 30 mcg weekly intramuscular IFNβ-1a (Avonex, Biogen Inc., Cambridge, MA) and 11 NC. This was an exploratory retrospective non-randomized two-arm observational preliminary study. All MS subjects were identified by chart review using the following inclusion criteria: RRMS [25], age 18 to 60 years, and an Expanded Disability Status Scale (EDSS) [26] score of 0–5. Patients were required to have a baseline, 1 year, and 2 year 3T MRI scan available. Clinical evaluation, including EDSS scoring and timed 25-foot walk (T25FW) [27], were assessed within 3 months of MRI by the treating neurologist at the Partners MS Center. When comparing groups on baseline characteristics, age and sex distributions were similar (Table 1). This study was approved by our institution’s research ethics committee.

**Table 1:** Baseline demographics and clinical data

	Multiple sclerosis	Normal controls	*p* value
Number of subjects	16	11	–
Age (years)	47.7 ± 7.5 (34–58)	43.1 ± 7.9 (30–53)	0.15*
Women, number (%)	14 (88 %)	8 (73 %)	0.37**
Disease duration (years)	15.0 ± 10.3 (4–35)	–	–
Expanded Disability Status Scale score, median (range)	1.5 (0–2.5)	–	–
Timed 25-foot walk (seconds)	4.6 ± 0.7 (3–6)	–	–
Time on interferon β-1a (months)	68.3 ± 59.9 (4–156)	–	–

Key: data are presented as mean ± standard deviation (range), unless otherwise indicated; disease duration = years from first symptoms; *two-sample *t*-test; **Fisher’s exact test

### MRI acquisition

**Fig. 1:**
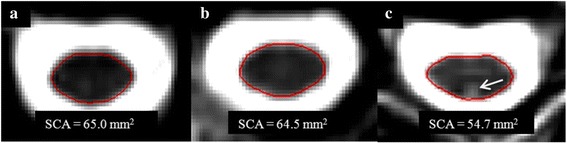
Sample images and segmentations results. Representative baseline cervical spinal cord T2-weighted axial images (taken at C3) from three patients with RRMS on interferon β-1a treatment. The corresponding region-of-interest (*red oval*) resulting from the semiautomated cord contouring tool is also shown. The normalized spinal cord area (SCA) for each patient, determined from C1-C5, is listed below the images. **a** A patient with a disease duration (DD) of 19.4 years, Expanded Disability Status Scale (EDSS) score of 2.0, and timed 25-foot walk (T25FW) of 4.0 s. **b** A patient with DD 4.4 years, EDSS 2.5, and T25FW 5.7 s. **c** A patient with DD 24.4 years, EDSS 1.5, T25FW 4.0 s, in whom successful spinal cord contouring was performed despite the presence of a T2 hyperintense spinal cord lesion (indicated with *arrow*)

All subjects underwent spinal cord 3T MRI using the same acquisition protocol and scanner (GE Signa, General Electric Healthcare, Milwaukee, WI). The scan protocol has been detailed previously [28]. 2D T2-weighted fast spin-echo imaging of the whole spinal cord was performed using 137–192 axial slices without gaps (TR/TE: 5933.34–6183.34/110.24–112.48 ms; voxel size 0.9375 × 0.9375 × 3 mm; number of signal averages: 2; field of view: 24 × 19 cm; scan duration: 18–38 min). Every patient, except for 4, had a TR of 6166.7 ms; the majority had a TE of 110.24 ms. We employed an 8 channel phased array coil, motion compensation, and interleaving, but no cardiac gating. Spinal cord imaging began superiorly at the base of the cerebellum. To ensure consistency between baseline and follow-up scans, patients were always positioned in the same orientation (head first with shoulders against the coil). We relied on T2-weighted images for cord volume determinations based on our previous work showing similar results between T1- and T2-derived spinal cord volume data, and no confounding effect of spinal cord T2 lesions on such determinations [29]. Scans were conducted at baseline, 1 and 2 years later. Sample images are shown in Fig. 1.

### MRI analysis

Image analysis was performed by a validated active surface method [2] using Jim (v.7, Xinapse Systems, West Bergholt, UK; www.xinapse.com). A marker was placed at the center of the spinal cord on the most superior axial slice of C1 in which the cerebellum was no longer visible. Additional markers were then placed in the center of the cord on every fifth slice until the bottom of C5 was reached. The active surface method was then applied to automatically produce regions-of-interests by distinguishing between the contour of the cord and the surrounding CSF. Total spinal cord volume was then calculated for C1-C5, from which the normalized cross-sectional spinal cord area (SCA) was derived by dividing by the total number of axial slices [30]. Analysis was performed by two trained observers who were blinded to both subject group and clinical data. Manual adjustments were applied to the final output maps as necessary. The high reliability of this method has already been established [29]. Sample segmentations are shown in Fig. 1. In addition, for descriptive purposes only and to assess to what extent patient’s had overt spinal cord pathology, the number of spinal cord lesions in the C1-C5 area, and the entire spinal cord was determined for each subject by the same trained observers.

### Statistical analysis

Baseline characteristics between groups were compared using two-sample t-tests and Fisher’s exact tests. One-sample t-tests and two-sample t-tests were employed to analyze the on-study change in SCA within each cohort and between MS and NC, respectively. Relationships between clinical characteristics and SCA were determined using Spearman correlations. A *p* < 0.05 was considered statistically significant; a *p* > 0.05 but <0.10 was considered a trend.

## Results

**Fig. 2:**
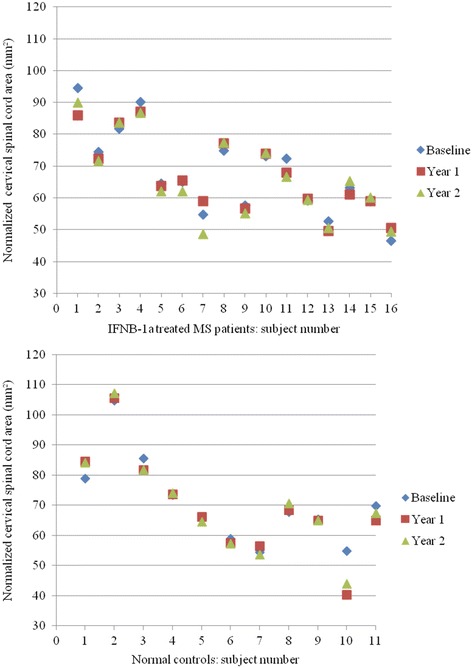
Spinal cord area over 2 years: individual subject results. Change in normalized spinal cord area over 2 years in each subject, including patients with relapsing-remitting multiple sclerosis (MS) on interferon β-1a treatment (IFNβ-1a) (*top*) and normal controls (*bottom*)

**Fig. 3:**
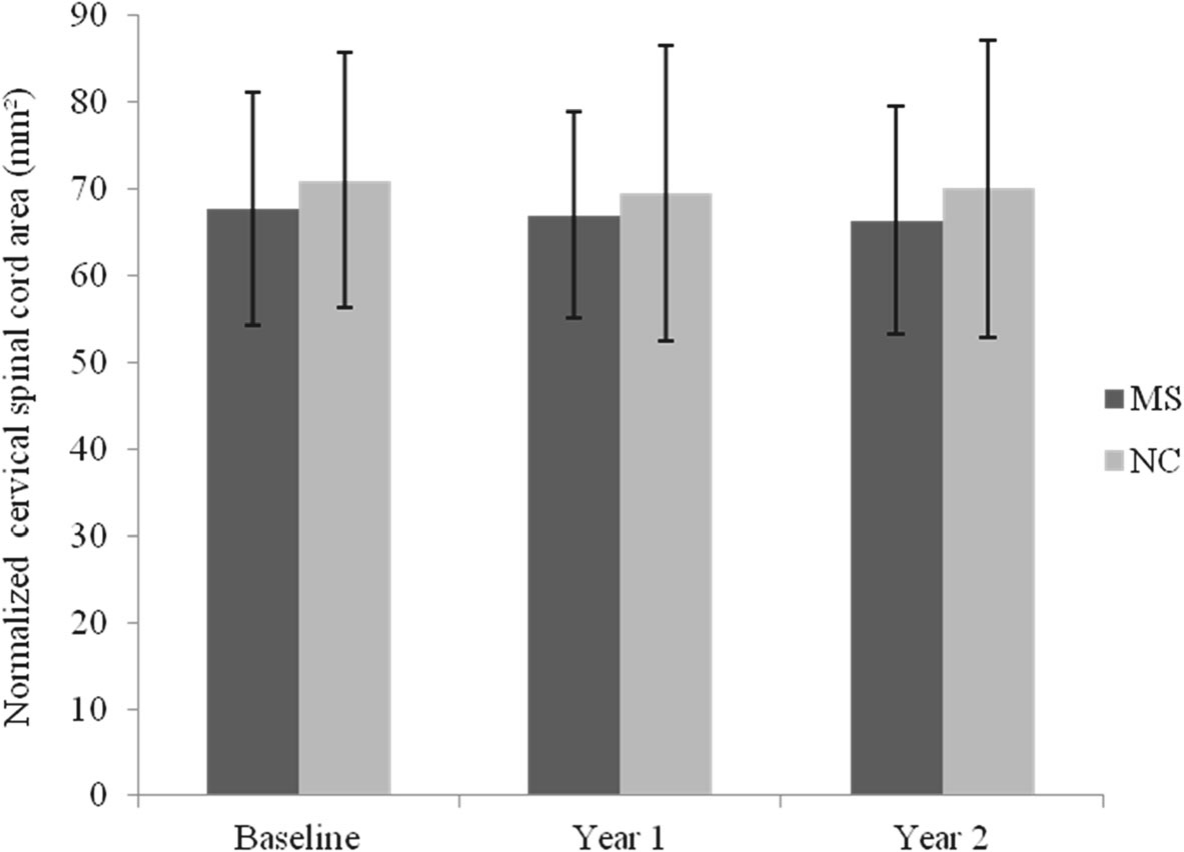
On-study change in spinal cord area: comparison of patient and control groups. Normalized spinal cord area (SCA) in patients with relapsing-remitting multiple sclerosis (MS) on interferon β-1a treatment vs. normal controls (NC) over 2 years. Means and standard deviation *error bars* are shown. SCA showed no change in the MS (*p* = 0.106) or NC (*p* = 0.524) groups over 2 years. Between group analysis indicated no differences in on-study SCA change (year 1 vs. baseline, *p* = 0.780; year 2 vs. year 1, *p* = 0.245; year 2 vs. baseline, *p* = 0.712). See also Table 3

**Fig. 4:**
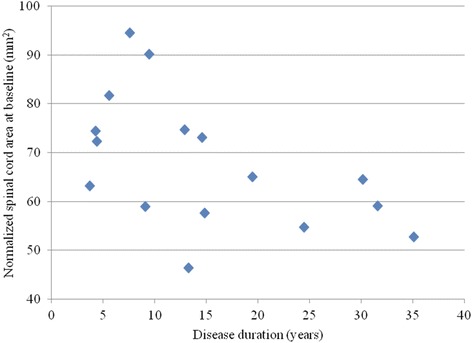
Spinal cord atrophy is related to disease duration. The scatter plot depicts the significant association in the MS group between decreasing normalized cervical spinal cord area and increasing disease duration at baseline (Spearman *r* = −0.518, *p* = 0.042)

The results are presented in Figs. 1, 2, 3, 4 and Tables 2, 3.

**Table 2:** Relationship between baseline cervical spinal cord area and clinical characteristics in the multiple sclerosis group

	r_s_	*p* value
Disease duration	−0.518	0.042*
Age	−0.291	0.273
Expanded Disability Status Scale score	0.170	0.529
Timed 25-foot walk	0.114	0.675

Key: r_s_ = Spearman’s correlation coefficient; *p < 0.05; see also Fig. 4

**Table 3:** Spinal cord area change over 2 years

	Multiple sclerosis			Normal controls			MS vs. NC	
		*p* value	% change		*p* value	% change		*p* value
Year 1 - Baseline	−0.673 (−2.463, 1.117)	0.435*	−0.41	−1.073 (−3.607, 1.460)	0.367*	−0.11	0.400 (−3.350, 2.549)	0.780**
Year 2 - Year 1	−0.710 (−2.482, 1.062)	0.406*	−0.55	0.486 (−0.768, 1.739)	0.408*	0.15	−1.196 (−0.875, 3.266)	0.245**
Year 2 - Baseline	−0.604 (−1.352, 0.144)	0.106*	−1.64	−0.360 (−1.576, 0.855)	0.524*	−0.78	−0.243 (−1.120, 1.607)	0.712**

Key: mean annualized change in normalized cervical spinal cord area in mm^2^ (95 % confidence interval) is reported; in addition, the median annualized percent change (%) is reported; the second (earlier) time point was subtracted from the first (later) time point listed; *MS* relapsing-remitting multiple sclerosis, *NC* normal controls, *one-sample *t*-test to assess the within group change; **two-sample *t*-test to assess the between group difference in changes

### Baseline comparisons

In the MS patients, a significant inverse correlation was observed between baseline SCA and disease duration (Spearman *r* = −0.518, *p* = 0.042, Table 2, Fig. 4). No significant associations were found between baseline SCA and baseline age, EDSS score, or T25FW (all *p* > 0.05, Table 2).

### On-study spinal cord change: within group comparisons

Considering the MS subgroup alone, no significant change was seen in SCA over the 2 year study period [mean annualized difference (95 % CI): −0.604 mm^2^ (−1.352, 0.144), *p* = 0.106]. Similarly, when assessing the on-study change in SCA within the NC cohort, no significant difference was detected over 2 years [−0.360 mm^2^ (−1.576, 0.855), *p* = 0.524] (Table 3, Figs. 2 and 3).

### On-study spinal cord area change: between group comparisons

When comparing the on-study changes in SCA between the MS and NC cohorts, no significant differences were detected [MS vs. NC mean annualized change difference (95 % CI) year 2 vs. baseline: −0.243 mm^2^ (−1.120, 1.607), *p* = 0.712]. Analysis comparing the change in SCA from baseline to year 1 and from year 1 to year 2 also indicated no significant differences between the two cohorts [year 1 vs. baseline: 0.400 mm^2^ (−3.350, 2.549), *p* = 0.780; year 2 vs. year 1: −1.196 mm^2^ (−0.875, 3.266), *p* = 0.245] (Table 3, Figs. 2 and 3).

### Spinal cord lesions

Spinal cord lesions in the C1-C5 area were detected in 10 subjects (63 %) from the MS cohort at baseline [mean number ± SD (range) per patient: 1.38 ± 1.45 (0–4) lesions] (Fig. 1). An analysis of baseline data showed that the MS subjects with cervical spinal cord lesions had a SCA of 63.22 ± 12.86 mm^2^, and the 6 MS subjects without lesions had a SCA of 75.2 ± 11.71 mm^2^. This difference was not statistically significant, but showed a trend (*p* = 0.082). The number of lesions per patient in the whole spinal cord was 3.00 ± 3.33 (0–12) at baseline, 3.69 ± 3.66 (0–14) at year 1, and 3.44 ± 2.61 (0–9) at year 2. Considering the whole spinal cord, most of the MS subjects had spinal cord lesions; only five patients (31 %) were free of lesions at baseline. This was reduced to 3 subjects (19 %) by year 2. No spinal cord lesions were detected in the NC group at any time point.

## Discussion

In this pilot study, we explored the effect of IFNβ-1a therapy on spinal cord atrophy over 2 years in patients with RRMS. Patients did not develop any atrophy over 2 years and had no difference in their spinal cord volume change as compared to healthy volunteers. This was a “real world” retrospective study without any comparison patient group, such as untreated patients. The sample size was small. Thus, the results should be interpreted with caution and the study design does not permit any strong conclusion regarding a treatment effect of IFNβ-1a. Nonetheless, the data provided here are valuable in that very few studies have examined spinal cord metrics under treatment with disease-modifying MS medications; most of the previous studies have focused on progressive rather than relapsing forms of the disease [8–14]. Thus, we provide a unique set of preliminary results that could serve as a basis for further studies on the role of spinal cord imaging in treatment monitoring in RRMS.

Spinal cord atrophy has been reported to occur in the early stages of MS, such as in patients with clinically isolated demyelinating syndromes or RRMS [1, 31–33]. However, the stage of the appearance of spinal cord atrophy is controversial; other studies have not confirmed these results and have contended that spinal cord atrophy most commonly develops in the later stages of RRMS or in progressive forms of the disease [2, 6, 9, 10, 30, 34]. In addition, transient changes such as inflammation and edema may increase spinal cord volume, particularly early in the MS disease course, and serve to offset or mask ongoing volume loss due to atrophy [35]. Thus, our study may have suffered from a diagnostic sensitivity bias in that the lack of spinal cord atrophy may have reflected the early disease stage of our patients rather than the effect of therapy. However, this was tempered by the observation that spinal cord lesions were quite common in our patients.

In the present study, we employed a highly reproducible semiautomated segmentation tool to measure spinal cord volume [2], which was normalized by our established method [30]. We applied this segmentation pipeline to 2D images, given their availability in this retrospective study and our previous demonstration that these images showed 1) sensitivity to disease-specific effects and 2) high reproducibility [29]. We did not have high-resolution 3D images available in this data set, which have been commonly used by several groups to effectively measure spinal cord volume [16, 36–38]. In addition, newer fully automated methods of contouring spinal cord volume have become available and may have relevance to MS [3, 6, 7], which we did not employ in this study. Thus, we cannot exclude the possibility that our technique, both on the basis of scan acquisition and post-processing methodology, may have lacked sensitivity to ongoing spinal cord atrophy in these patients, irrespective of a drug treatment effect.

Although we had a small sample size, we chose to test the relationship between SCA and clinical status in the MS group at baseline, to explore the validity of our results. We failed to find any significant correlations between SCA and measures of overall physical disability (EDSS score) or ambulatory function (T25FW). However, we showed that SCA significantly correlated inversely with disease duration, indicating that spinal cord atrophy was linked to advancing disease duration. Previous studies have shown inconsistent results regarding the relationship between spinal cord atrophy and clinical status in MS. Some studies have shown a correlation between spinal cord atrophy and advancing EDSS score, [2, 4, 6, 7, 10, 15, 17, 18, 29, 30, 33, 34, 39], while others have not [1, 8, 10]. A significant relationship between spinal cord atrophy and ambulatory dysfunction on the T25FW has been shown in some [4, 6, 30] but not all studies [18, 29]. Furthermore, a growing body of evidence indicates that spinal cord-disability relationships are more strongly present in patients with advanced disability and progressive stages of the disease [1, 4, 8, 9, 18, 34, 39], the stage at which spinal cord atrophy is most commonly seen [2, 6, 10, 30, 34]. Therefore, in addition to the small sample size, our inability to show a relationship between SCA and disability measures may reflect the restricted range of our mildly disabled relapsing-remitting stage patients. Nonetheless, the link we showed between SCA and disease duration is consistent with previous work [7, 33, 34] and provides some reassurance of the validity of the SCA measure employed in our study.

## Conclusion

Established IFNβ-1a therapy was not associated with ongoing spinal cord atrophy or any difference in the rate of spinal cord volume change in RRMS compared to NC over 2 years. These results may reflect a treatment effect. However, due to sample size and study design, these results should be considered preliminary and await confirmation in larger prospective studies.

## Abbreviations

EDSS: Expanded Disability Status Scale
IFNβ-1a: Interferon beta-1a
MS: Multiple sclerosis
NC: Normal control
RR: Relapsing-remitting
SCA: Normalized spinal cord area
T25FW: Timed 25-foot walk
